# Retropharyngeal Lymph Node Involvement in Oropharyngeal Carcinoma: Impact upon Risk of Distant Metastases and Survival Outcomes

**DOI:** 10.3390/cancers12010083

**Published:** 2019-12-29

**Authors:** Zsuzsanna Iyizoba-Ebozue, Louise J. Murray, Moses Arunsingh, Karen E. Dyker, Sriram Vaidyanathan, Andrew F. Scarsbrook, Robin J. D. Prestwich

**Affiliations:** 1 Department of Clinical Oncology, Leeds Cancer Centre, Leeds LS97TF, UK; zsuzsanna.iyizoba@nhs.net (Z.I.-E.); louise.murray8@nhs.net (L.J.M.); 85moses@gmail.com (M.A.); karen.dyker@nhs.net (K.E.D.); 2 Leeds Institute of Medical Research at St James’s, University of Leeds, Leeds LS97TF, UK; a.scarsbrook@nhs.net; 3 Department of Nuclear Medicine and Radiology, Leeds Cancer Centre, Leeds LS97TF, UK; svaidyanathan@nhs.net

**Keywords:** oropharynx cancer, radiotherapy, chemotherapy, lymph node, retropharyngeal, distant metastases, survival

## Abstract

The influence of retropharyngeal lymph node (RPLN) involvement on prognosis in oropharyngeal carcinoma remains poorly defined. The aim of this study was to assess the impact of RPLN involvement upon outcomes. A single-centre retrospective analysis of 402 patients with oropharyngeal carcinoma treated nonsurgically between 2010 and 2017 was performed. All had a baseline 2-[fluorine-18]-fluoro-2-deoxy-d-glucose (FDG) PET-CT and contrast-enhanced MRI and/or CT. RPLN status was determined by radiology review of cases with reported abnormal RPLN. Multivariate backwards logistic regression was used to examine impact on outcomes of factors. Abnormal RPLNs were identified in 40/402 (10%) of patients. Median follow up was 42.9 months. RPLN involvement was associated with inferior 3 year outcomes for overall survival (OS) (67.1% vs. 79.1%, *p* = 0.006) and distant metastases-free survival (DMFS) (73.9% versus 88.0%, *p* = 0.011), with no significant difference in local control (81.6% vs. 87.7%, *p* = 0.154) or regional control (80.7% vs. 85.4%, *p* = 0.252). On multivariate analysis abnormal RPLN, no concurrent chemotherapy and ongoing smoking were associated with inferior DMFS and OS, while advanced T stage was also associated with inferior OS. In summary, RPLN involvement, present in 10% of patients, was an independent prognostic factor for the development of distant disease failure translating into inferior OS. These findings need confirmation in future studies.

## 1. Introduction

Nonsurgical treatment with radiotherapy and chemotherapy is a standard of care for the management of oropharyngeal carcinoma [1]. Human papilloma virus (HPV)-related oropharyngeal carcinoma has a more favourable prognosis than non-HPV-related disease [1,2]. There has been considerable interest in exploring “de-escalation” strategies for better disease prognosis in view of the substantial toxicity of chemoradiotherapy [3,4,5,6]. The negative results of recent de-escalation studies seeking to replace concurrent cisplatin with cetuximab [3,6] emphasise the importance of accurate identification of patients with low-risk disease. The development of distant metastases remains a concern, even in apparently favourable prognosis disease [7]. Rates of distant metastases appear similar for HPV-positive and -negative disease, with controversy over whether HPV status influences the pattern of distant metastases [7,8,9,10].

Regional lymph node metastases from oropharyngeal squamous cell carcinoma can occur in cervical lymph nodes and retropharyngeal lymph node (RPLN) beds [11]. The retropharyngeal space extends from the skull base to the level of the C3 vertebra caudally, posterior to the constrictor muscles and anterior to prevertebral fascia [11]. RPLN involvement has been found to be limited to the lateral retropharyngeal compartment [12,13,14,15,16] and this is reflected in contouring guidelines [17]. The difficulty in surgical access to the retropharyngeal area limits data on the incidence of RPLN metastases [11]. Surgical series are small and restricted to highly selected operable patients [18,19], hence limiting their applicability to the wider group of oropharyngeal carcinoma. Imaging studies have reported a very variable incidence of RPLN involvement, with larger series reporting RPLN involvement in 9–21% of oropharyngeal carcinomas [12,13,14,20,21,22,23].

The American Joint Committee on Cancer (AJCC) TNM oropharynx staging system [24] does not include RPLN as a site of regional lymph nodes and RPLNs are not included as an independent prognostic factor. Recent treatment de-escalation studies have not excluded patients with RPLN involvement [3,6]. There is limited data regarding the prognostic influence of radiological RPLN involvement in oropharyngeal carcinoma. Some studies have suggested that RPLN involvement in patients with oropharyngeal carcinoma is associated with inferior survival and higher risk of development of distant metastatic disease [13,15]. The prognostic implications of RPLN in patients with HPV-related oropharyngeal disease are controversial, with some, but not all, studies suggesting a negative relationship [12,15,20]. Elucidating the prognostic impact of RPLN involvement is important to accurately stratify risk in oropharyngeal carcinoma. If RPLN involvement predicts inferior outcomes, this should be considered as an exclusion criterion from studies investigating treatment deintensification.

We have recently reported on the incidence and patterns of RPLN involvement in oropharyngeal carcinoma in a series of 402 patients treated with chemoradiotherapy who had baseline 2-[fluorine-18]-fluoro-2-deoxy-d-glucose (FDG) PET-CT along with an MRI and/or contrast-enhanced CT [16]. The purpose of this study was to analyse the impact of RPLN upon survival and disease outcomes.

## 2. Results

### 2.1. Whole Cohort

Table 1 summarises baseline demographics, tumour characteristics and treatment for the cohort of 402 patients with oropharyngeal carcinoma. Among patients, 382/402 (95.0%) had stage III/IV disease according to the AJCC TNM seventh edition [25], 76/402 (18.9%) received radiotherapy alone, 1/402 (0.2%) received induction chemotherapy (ICT) followed by radiotherapy, 17/402 (4.2%) received induction chemotherapy followed by concurrent chemotherapy and 308/402 (76.6%) received concurrent chemotherapy without induction. Median follow up was 42.9 months (95% confidence interval: 39.9–45.9). For the whole cohort of 402 patients, 3 year overall survival (OS), local control, regional control and distant metastasis-free survival (DMFS) were 77.9%, 87.0%, 84.7% and 86.6%, respectively. In total, disease progression occurred in 99/402 (24.6%) patients. This included 48 patients with local progression, 55 with regional lymph node progression and 48 who developed distant metastases.

All patients had a baseline staging PET-CT and also diagnostic imaging with MRI (n = 339) and/or contrast-enhanced CT (n = 83). Details of the identification and imaging features of RPLNs have been previously reported [16] and are summarised here. Based upon review of clinical records, radiology reports and radiotherapy plans for a total of 43/402 patients with RPLN were identified. Following imaging review by a radiologist, 40/402 (10%) patients were classified as having abnormal RPLN; 37/40 RPLN were FDG-avid on PET-CT (median SUV_max_ 6.8 (range 3–26.3)) and 35/37 patients had a corresponding abnormal RPLN on diagnostic MRI and/or contrast-enhanced CT. Three patients had non-FDG avid abnormal RPLN identified on MRI (short axis: 5–9 mm). Abnormal RPLNs were located in the ipsilateral retropharyngeal area in only 32/40 patients, bilateral retropharyngeal regions in 5/40 patients and the contralateral retropharyngeal region in only 3/40 patients.

**Figure 1:**
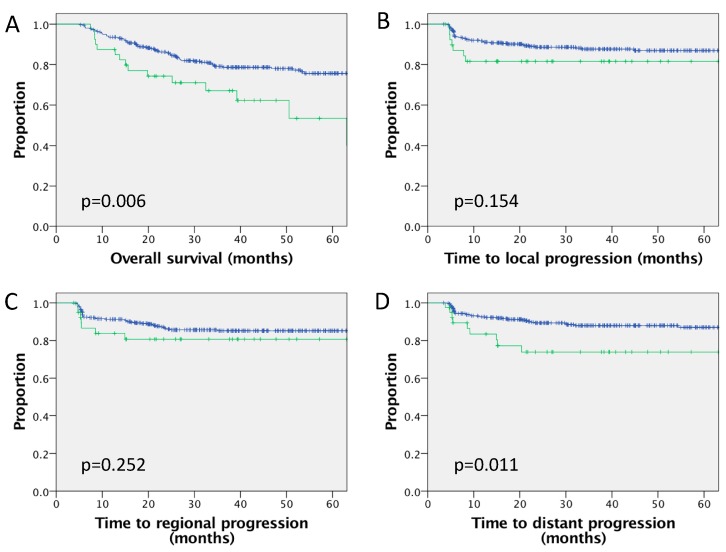
Survival outcomes for a cohort of 402 patients comparing outcomes with or without RPLN involvement. Kaplan–Meier curves for (**A**) overall survival, (**B**) local (primary tumour) progression, (**C**) regional (lymph node) progression and (**D**) distant metastatic progression. RPLN negative (n = 362) in blue and RPLN positive (n = 40) in green.

RPLN involvement (40/402 (10%) of patients) was associated with inferior 3 year outcomes for OS (67.1% versus 79.1%, *p* = 0.006) and DMFS (73.9% versus 88.0%, *p* = 0.011), with no significant difference in local control (81.6% versus 87.7%, *p* = 0.154) or regional control (80.7% versus 85.4%, *p* = 0.252). Figure 1 provides the Kaplan–Meier survival curves illustrating outcomes for patients with or without RPLN involvement. Table 2 summarises the multivariate analysis of the whole cohort (not using p16 status as a factor due to missing data). RPLN involvement, no concurrent chemotherapy, ongoing smoking and more advanced T stage were associated with inferior OS. RPLN involvement, no concurrent chemotherapy and ongoing smoking were independently associated with inferior DMFS.

### 2.2. Cohort with p16 Status

**Figure 2:**
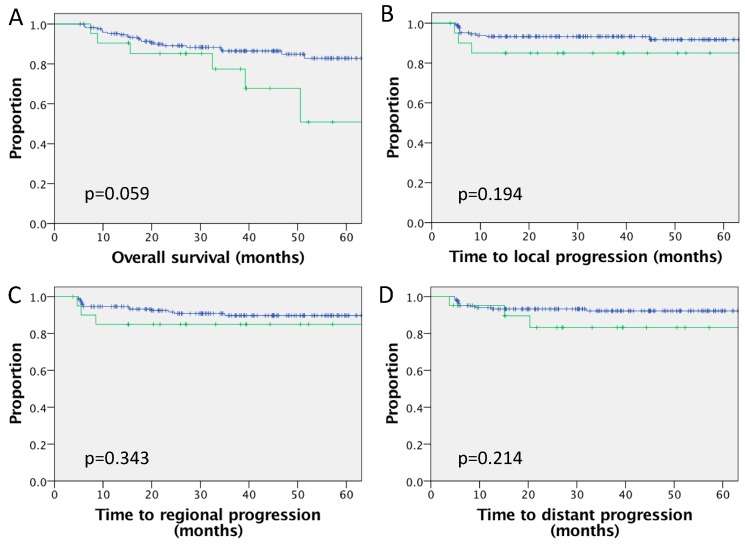
Survival outcomes for cohort of p16-positive (n = 192) disease comparing patients with or without RPLN involvement. Kaplan–Meier curves for (**A**) overall survival, (**B**) local (primary tumour) progression, (**C**) regional (lymph node) progression and (**D**) distant metastatic progression. RPLN negative (n = 171) in blue and RPLN positive (n = 21) in green.

Routine p16 testing was only introduced into clinical practice partway through this patient cohort and 226/402 (56.2%) patients had p16 status available. Among patients, 192/226 (85.0%) with p16 status available were p16 positive, and 21/192 (10.9%) of patients with p16-positive tumours had abnormal RPLN. Outcomes for the 192 patients with proven p16-positive disease according to RPLN status are shown in Figure 2, with a nonsignificant trend for inferior outcomes for p16-positive patients with involved RPLN compared to those without. Three-year outcomes for patients with abnormal RPLN versus those without abnormal RPLN were OS (77.4% versus 86.5%, *p* = 0.059), DMFS (83.2% versus 92.3%, *p* = 0.214), local control (85.0% versus 93.3%, *p* = 0.194) and regional control (85.0% versus 89.8%, *p* = 0.343). Only 34 patients had proven p16-negative disease, of which 6/34 (17.6%) had abnormal RPLN. Three-year outcomes for patients with known p16-negative disease with RPLN were OS (66.7% versus 64.7%, *p* = 0.579), DMFS (62.5% versus 81.3%, *p* = 0.292), local control (83.3% versus 69.8%, *p* = 0.858) or regional control (60.0% versus 89.8%, *p* = 0.390). Univariable analysis is shown in Table 3. For the subgroup of p16-positive patients, the N stage was also converted to AJCC TNM8 [25]; there was no significant association between N stage and OS or DMFS (for OS, N1 vs. N0, *p* = 0.905; N2 vs. N0, *p* = 0.900; N3 vs. N0, *p* = 0.891; and for DMFS OS, N1 vs. N0, *p* = 0.940; N2 vs. N0, *p* = 0.938; N3 vs. N0, *p* = 0.931). On multivariate analysis of the 226 patients with known p16 status (Table 3), ongoing smoking, p16 negative and more advanced T stage were associated with inferior OS. T stage and p16 status were independently associated with inferior DMFS.

## 3. Discussion

This study analysed the impact of RPLN involvement on outcomes in a large retrospective cohort of patients with oropharyngeal carcinoma treated with chemoradiotherapy. This is the largest reported series other than the two overlapping publications from the MD Anderson Cancer Centre [13,20]. The accurate identification of RPLN involvement is critical to this analysis. Determination of abnormal RPLN was based on established radiological criteria, including size, necrosis and FDG activity [20,23,26,27,28] by a dual-certified radiologist and nuclear medicine physician. Among patients, 84% in our series had a diagnostic MRI; MRI has been shown to be superior to contrast-enhanced CT for identification of RPLN metastases [11,29,30]. The most sensitive imaging modality for the identification of lymph node involvement in head and neck cancer is FDG PET-CT [31,32]. In one small series of patients who underwent retropharyngeal dissections, all patients who had evidence of FDG-avid RPLN on staging PET-CT had pathological RPLN involvement [19]. The imaging used in prior reported series to identify RPLN (Table 4) had varied widely and would be expected to impact upon the rates of RPLN identification. In several prior series examining the incidence of RPLN in oropharyngeal carcinoma, very limited numbers of patients had PET-CT staging [12,13,20,21]. In contrast, in our series, all patients had a staging PET-CT scan in addition to MRI and/or CT. Combining PET-CT with cross-sectional anatomical imaging with MRI or CT has been shown to increase the accuracy of RPLN detection [22,33]. Ours is the largest reported series in which all patients had a staging PET-CT; a considerably higher proportion of patients also had had a diagnostic MRI by comparison with other similar series (Table 4). In contrast, in the large series reported by Gunn et al. [13], only 29% of patients had a PET and 9% MRI with the majority of RPLN identified on the basis of CT alone; similarly, in the follow-up by Lin et al. [20], the majority of RPLN were identified only by CT.

A limited number of prior studies have reported RPLN involvement in oropharyngeal carcinoma, with a variable incidence (Table 4) [12,13,14,20,21,22,23,34]. In our series of 402 patients, the rate of radiological RPLN involvement was 10%. This is very similar to the rate of RPLN involvement in the largest reported series from the MD Anderson Cancer Centre of 981 patients [13] of 10% and their partially overlapping series of 781 HPV-positive patients with a rate of 9%. Other reported series [12,14,15,21,22] are considerably smaller than these of our series, which may at least partially explain the variability in the reported rates of RPLN involvement.

In the current analysis of the whole cohort, RPLN involvement was associated with significantly inferior OS in multivariate analysis. Although rates of local or regional disease control were numerically inferior in patients with RPLN involvement, differences were small and nonstatistically significant. Based upon the significant association of RPLN involvement with inferior DMFS in the absence of an association with local or regional control, it can be hypothesised that the impact upon OS of RPLN involvement is mediated via an increased risk of distant metastases (Figure 1), with which RPLN involvement was also independently associated in multivariate analysis.

HPV status is a key prognostic factor in oropharyngeal carcinoma [1]. Routine testing with p16 (as a surrogate for HPV status) was only introduced into routine practice partway through the period of this study and is only available for 56% of these patients. In patients tested, the rate of p16-positive disease is high at 85%; therefore, it is likely that the prevalence of HPV-related disease in the overall cohort is high. Statistical analysis of the subgroup with proven p16-positive disease is limited by the size of the subgroup and the number of patients with abnormal RPLN (n = 21/192). Within this p16-positive subgroup, a similar trend was observed to that in the overall cohort, with a trend toward inferior outcomes for patients with RPLN involvement (Figure 2), although differences were not statistically significant and RPLN involvement did not remain in the final model of multivariate analysis.

There are only a small number of series which have evaluated the prognostic impact of RPLN involvement; these are summarised in Table 4. The largest series of 981 patients from the MD Anderson Cancer Centre is based on patients treated between 2001 and 2007 and HPV data are not available [13]. This series reported that RPLN involvement was associated with inferior OS along with lower rates of local and distant disease control but not regional control in multivariate analysis. In this series, treatment approaches were heterogenous, including patients receiving short palliative radiotherapy schedules. In contrast, in our series, all patients received curative-intent treatment. The results from our analysis confirm the association of RPLN with DMFS and OS but, in contrast, did not demonstrate an association with local control. In a follow-up report focusing on 739 patients with HPV-positive and node-positive disease treated between 2004 and 2013 [20] (a partially overlapping series of patients with their original study), the presence of abnormal RPLN was associated with inferior OS and DMFS but not local or regional control in univariate analysis, although differences were not maintained in multivariate analysis. These data appear to support the finding in our series of the lack of association between RPLN and local control. The work of Lin et al. [20] and our data, therefore, suggest that although RPLN as a single variable was associated with inferior OS/DMFS in HPV-positive patients, the overall more favourable prognosis of HPV-positive disease made it difficult to detect statistically significant differences in relation to RPLN. In a series of 185 patients with HPV-positive disease from the University of Michigan [15], radiological RPLN involvement was associated with inferior OS and DMFS, with no difference in local or regional control. In contrast with the results of Lin et al. [20] and our subgroup of 192 patients with p16-positive disease, this association was maintained in multivariate analysis. The authors concluded that RPLN were an independent risk factor for the development of distant metastases and that this led to inferior failure-free and OS outcomes. A recent analysis of HPV-positive patients who were stage 1 according to the recent AJCC Cancer Staging, eighth edition [24] reported an association between RPLN involvement and distant metastases after adjusting for radiological extracapsular extension [35]. Other smaller studies [12,22] have shown trends toward inferior outcomes in patients with involved RPLN without significance in univariate [12] or multivariate analysis [22].

Overall, based upon our results and published data (Table 4), it appears that RPLN involvement is a risk factor particularly for the development of distant metastatic disease and, consequently, inferior overall outcomes whilst not being consistently predictive of locoregional control. In HPV-positive patients’, similar trends were observed but differences in disease outcomes were smaller, likely relating to lower event rates, and statistical significance between RPLN involvement and outcomes was more difficult to demonstrate.

The limitations of this series include the retrospective nature of the analysis and the heterogeneity of treatment, including the use of induction and/or concurrent chemotherapy. Only 4% of patients were treated with induction chemotherapy, with no significant difference in the rate of RPLN involvement between those receiving/not receiving induction chemotherapy; therefore, it is not possible to draw any conclusions regarding the potential value of induction chemotherapy in the presence of RPLN disease. The absence of p16 status for 44% of patients limits the ability of analysis to detect significant associations of RPLN involvement with outcomes in both the p16-positive and -negative subgroups. However, in patients with known p16 status, the rate of p16 positivity was 85%, with no statistical difference in the rate of RPLN involvement in patients without p16 status or p16+ve or p16−ve disease; this implies that analysis of the overall cohort remains informative. In addition, only a small number of patients had early stage disease (according to the TNM seventh edition classification in use at the time of treatment) and the applicability to this subgroup is limited. It was only possible to classify smoking history into non-, former and current smoking. There was insufficient detail in clinical notes to accurately assign the number of pack/years smoked which would be regarded as a gold standard for smoking history. We have previously reported on patterns of RPLN (ipsilateral, contralateral and bilateral) [16]; only a small number of patients have bilateral or contralateral-only lymph nodes precluding any analysis to determine whether these patterns have any prognostic implications beyond RPLN involvement per se.

Overall, based upon our results and published data (Table 4), it appears that RPLN involvement is a risk factor particularly for the development of distant metastatic disease and, consequently, inferior overall outcomes whilst not being consistently predictive of locoregional control. In HPV-positive patients, similar trends were observed but differences in disease outcomes were smaller, likely related to lower event rates, and statistical significance between RPLN involvement and outcomes is more difficult to demonstrate.

## 4. Materials and Methods

### 4.1. Study Group

This was a single-centre retrospective analysis at Leeds Cancer Centre. An electronic database was used to identify patients receiving chemoradiotherapy between January 2010 and June 2017. Inclusion criteria for the analysis were oropharyngeal squamous cell carcinoma, curative treatment with radiotherapy ± chemoradiotherapy, pretreatment staging FDG PET-CT and either contrast-enhanced MRI or CT of the neck or both. Exclusion criteria were prior radiotherapy or therapeutic surgery prior to baseline imaging. Electronic patient records were used to extract demographic and clinical data. Staging was documented according to the AJCC TNM staging, seventh edition in use during the study period [25]. Analysis of p16 immunohistochemistry was only implemented partway through this study period into routine clinical practice (due to the absence of data that p16 status should impact upon treatment). Scoring of p16 immunohistochemistry status was performed using a threshold of strong and diffuse nuclear and cytoplasmic staining in ≥70% of the tumour [36].

### 4.2. Identification of Retropharyngeal Lymph Nodes

Involvement of RPLN was determined by review of electronic records including imaging reports and also by review of LN gross tumour volumes (GTVs) in radiotherapy plans. Subsequently, all imaging for patients with suspected RPLN involvement was reviewed by a dual-certified radiologist and nuclear medicine physician with 15 years of experience of head and neck imaging. Normal lateral RPLNs are smaller than 4–4.5 mm in short axis [11]. Radiological criteria were used to identify involved RPLN and were defined for the study as short axis ≥ 5 mm, necrosis [20,26,27] and/or abnormal tracer uptake on PET-CT [23,28].

### 4.3. Chemotherapy

ICT was used for selected patients based upon clinician preference and patient and disease factors. The induction chemotherapy used was either TPF (docetaxel 75 mg/m^2^, cisplatin 75 mg/m^2^ and 5-flurouracil (5-FU) 750 mg/m^2^, days 2–5) [37] or PF (cisplatin 80 mg/m^2^ and 5-FU 800 mg/m^2^) [38]. Standard concurrent chemotherapy was cisplatin 100 mg/m^2^ (days 1 and 29). In the event of a contraindication to cisplatin, carboplatin area under the curve (AUC) 4 was substituted.

### 4.4. Radiotherapy

Radiotherapy techniques and the approach to target delineation changed several times during the study period of 2010–2017 with a conformal 3D-CT planned technique used in the early part of the time period, followed by 5–7 angle step-and-shoot intensity modulated radiotherapy (IMRT) and then replaced by volumetric modulated arc therapy (VMAT). The conformal 3D-CT planned technique has been previously described and used a compartmental approach to target volume delineation with the whole oropharynx and whole involved lymph node levels included within high dose volumes [39]. During the initial phase of IMRT implementation, a compartmental approach to target delineation was adopted based upon that used in the PARSPORT randomised trial [40,41]. This involved a primary tumour clinical target volume (CTV) including at least gross tumour volume (GTV) +10 mm, modified to anatomical boundaries and also encompassing the whole involved anatomical compartment (e.g., whole oropharynx, with the high dose nodal CTV including whole involved nodal levels). For some cases from 2014, at the discretion of the treating clinician and then routinely from 2016, a geometric (rather than routine inclusion of the whole oropharynx) approach to outlining was used with a high dose CTV based upon primary tumour and involved lymph nodes +10 mm and lymph node levels treated within elective dose CTVs. The lymph node target routinely included levels 1b–V in the node-positive neck; nodal levels in a node-negative neck were selectively irradiated depending upon tumour site and disease extent according to published recommendations [42]. Our approach to elective irradiation of RPLN has changed over the time period of this study; in the initial part of the study, RPLNs were treated to the skull base in line with consensus guidelines [42], and the superior border was subsequently reduced to the top of hard palate/C1 in line with the consensus document [17]. Prior to 2014, bilateral RPLNs were routinely treated and, subsequently, contralateral RPLN in patients with no contralateral lymph node disease was spared at clinician discretion based upon emerging data [43]. Pharyngeal constrictor muscles were not outlined as an organ at risk and no deliberate attempt was made to minimise the dose to those regions.

The planning target volume (PTV) was created by autoexpansion of the CTV by 4 mm [16]. Standard dose fractionations were 70 Gy in 35 fractions over 7 weeks or an option of 65 Gy in 30 fractions over 6 weeks (for patients treated without concurrent chemotherapy, with lower doses to the prophylactic dose regions (54–63 Gy in 30–35 fractions over 6–7 weeks).

### 4.5. Response Assessment and Follow-Up

Response was routinely assessed 4 months after treatment by clinical examination, nasoendoscopy (if indicated) and FDG PET-CT; examination under anaesthetic and biopsies were performed on clinical discretion following response assessment. Patients were routinely followed up for at least 5 years prior to discharge.

### 4.6. Statistical Analysis

Analysis was performed using IBM SPSS Statistics, Version 24 (Armonk, NY, USA: IBM Corp.). Chi-squared and Mann–Whitney *U* tests were used to investigate potential differences between clinical variables and the presence or absence of RPLN. Follow up and survival outcomes were calculated from the final day of radiotherapy. OS, progression-free survival (PFS), local control, regional control and DMFS were considered as endpoints and were calculated using the Kaplan–Meier method. Univariate and multivariate analyses were performed using Cox proportional hazards with backward likelihood ratios. Variables included age, sex, T and N stage, use of concurrent chemotherapy, smoking status, presence of RPLN and p16 status. Factors with *p* < 0.2 in univariate analysis were included in the multivariate analysis. Multivariate analysis was performed separately with and without p16 as a factor given the smaller proportion of patients with p16 status available. A *p*-value of <0.05 was considered statistically significant.

## 5. Conclusions

In summary, radiological involvement of RPLN in patients with oropharyngeal carcinoma is an independent factor associated with a higher risk of development of distant metastases and inferior OS. This raises the possibility that patients with RPLN involvement may not be suitable candidates for deintensification of systemic therapy and may be considered as a prognostic marker to identify subgroups of patients at higher risk of development of distant disease. These findings require confirmation in prospective studies.

## Figures and Tables

**Table 1:** Patient, disease and treatment characteristics.

Characteristics	Total Cohort (Column %) n = 402	RP Lymph Node Negative (Column %) n = 362	RP Lymph Node Positive (Column %) n = 40	*p*-Value
Age				0.297
Median (range)	57 (24–84)	57 (24–84)	56 (37–73)	
Gender				0.393
Male	310 (77.1)	277 (76.5)	33 (82.5)	
Female	92 (22.9)	85 (23.5)	7 (17.5)	
Smoking				0.868
Never	114 (28.4)	103 (28.5)	11 (27.5)	
Former	137 (34.1)	124 (34.3)	13 (32.5)	
Current	133 (33.1)	118 (32.6)	15 (37.5)	
Not recorded	18 (4.5)	17 (4.7)	1 (2.5)	
p16 status				0.266
Positive	192 (47.8)	171 (47.2)	21 (52.5)	
Negative	34 (8.5)	28 (7.7)	6 (15.0)	
Unknown	176 (43.8)	163 (45.0)	13 (32.5)	
Tumour subsite				0.002 *
Tonsil	241 (60.0)	219 (60.5)	22 (55.0)	
Base of Tongue	135 (33.6)	123 (34.0)	12 (30.0)	
Vallecula	9 (2.2)	9 (2.5)	0 (0)	
Postpharyngeal	5 (1.2)	4 (11.0)	1 (2.5)	
Soft palate	12 (3.0)	7 (1.9)	5 (12.5)	
T stage				0.194
T1	85 (21.1)	78 (21.5)	7 (17.5)	
T2	168 (41.8)	156 (43.1)	12 (30.0)	
T3	76 (18.9)	66 (18.2)	10 (25.0)	
T4	73 (18.2)	62 (17.1)	11 (27.5)	
N stage (AJCC 7th)				**0.005**
N0	41 (10.2)	39 (10.8)	1 (5.0)	
N1	45 (11.2)	44 (12.2)	2 (2.5)	
N2a	30 (7.5)	30 (8.3)	0 (0)	
N2b	199 (57.0)	179 (49.4)	20 (50.0)	
N2c	74 (18.4)	59 (16.3)	15 (37.5)	
N3	13 (3.2)	11 (3.0)	2 (5.0)	
Stage (AJCC 7th)				0.207
1	1 (0.2)	1 (0.3)	0 (0)	
2	19 (4.7)	17 (4.7)	2 (5.0)	
3	47 (11.7)	47 (13.0)	0 (0)	
4	335 (83.3)	297 (82.0)	38 (95.0)	
Histological grade				0.061
Well	3 (0.8)	3 (0.8)	0 (0)	
Moderate	74 (18.4)	62 (17.1)	12 (30.0)	
Poor	297 (73.9)	272 (75.1)	25 (62.5)	
Unclassified	28 (6.9)	25 (6.9)	3 (7.5)	
Induction chemotherapy				0.524
No	384 (95.6)	345 (95.3)	39 (97.5)	
PF	1 (0.2)	1 (0.3)	0 (0)	
TPF	17 (4.2)	16 (4.4)	1 (2.5)	
Concurrent chemotherapy				0.707
No	77 (19.2)	70 (19.3)	7 (17.5)	
Yes	325 (80.8)	292 (80.7)	33 (82.5)	

* Analysis comparing three groups (1. tonsil, 2. base of tongue and vallecula, 3. soft palate and posterior pharyngeal wall). AJCC = American Joint Committee on Cancer; PF = platinum/5-fluorouracil; RP = retropharyngeal; TPF = docetaxel, cisplatin, 5-fluorouracil. Significant *p*-values highlighted in bold.

**Table 2:** Predictors of distant metastases-free survival (DMFS) and overall survival (OS) in the whole cohort (n = 402, including 40 patients with RPLN involvement). Univariate analysis and multivariate Cox proportional hazards analysis using backwards likelihood ratios of established prognostic factors. Factors included in analysis were age, sex, T and N stage, presence of RPLN, use of concurrent chemotherapy and smoking status.

**Overall Survival**						
	**Univariate**			Multivariate *		
	**HR**	**95% CI**	***p*-Value**	**HR**	**95% CI**	***p*-Value**
Age	1.02	1.00–1.05	**0.022**			
Sex			0.780			
T stage						
T2 vs. T1	2.27	1.05–4.88	**0.037**	2.30	1.02–5.20	0.046
T3 vs. T1	2.91	1.28–6.64	**0.011**	2.40	1.00–5.79	0.051
T4 vs. T1	4.62	2.11–10.43	**<0.001**	3.64	1.58–8.43	0.003
N stage						
N1 vs. N0	0.37	0.11–1.19	**0.094**			
N2a vs. N0			0.230			
N2b vs. N0			0.991			
N2c vs. N0			0.344			
N3 vs. N0	2.37	0.84–6.65	**0.103**			
RPLN status (+ve vs. −ve)	2.13	1.22–3.71	**0.008**	2.00	1.13–3.54	0.018
Concurrent chemotherapy (no vs. yes)	2.17	1.39–3.38	**0.001**	2.02	1.29–3.19	0.002
Smoking						
Former vs. never	1.92	0.97–3.83	**0.063**			
Current vs. never	4.23	2.27–8.00	**<0.001**	3.58	1.89–6.77	<0.001
**Distant Metastasis–Free Survival**						
	**Univariate**			Multivariate *		
Age			0.513			
Sex			0.314			
T stage						
T2 vs. T1	2.01	0.75–5.38	**0.166**			
T3 vs. T1	2.44	0.83–7.14	**0.104**			
T4 vs. T1	3.84	1.38–10.7	**0.010**			
N stage						
N1 vs. N0			0.582			
N2a vs. N0			0.792			
N2b vs. N0	2.82	0.67–11.88	**0.157**			
N2c vs. N0	3.76	0.85–11.67	**0.081**			
N3 vs. N0	4.13	0.58–2.32	**0.156**			
RPLN status (+ve vs. −ve)	2.48	1.20–5.13	**0.014**	2.68	1.29–5.57	0.008
Concurrent chemotherapy (no vs. yes)	1.94	1.04–3.62	**0.037**	1.94	1.03–3.65	0.039
Smoking						
Former vs. never	2.66	0.79–5.37	**0.138**			
Current vs. never	4.36	1.79–10.55	**0.001**	4.17	1.71–10.16	0.002

* Factors with *p* < 0.2 in univariate analysis included in multivariate model; *p*-values for these are shown in bold. Hazard ratio and confidence interval shown for factors *p* < 0.2 carried forward into multivariate model. If factors were dropped as nonsignificant from multivariable analysis using backwards likelihood ratios, there were no *p*-values or HR to report. HR = hazard ratio; CI = confidence interval; RPLN = retropharyngeal lymph node.

**Table 3:** Predictors of DMFS and OS in cohort with known p16 status (n = 226, including 27 patients with RPLN involvement): Cox proportional hazards regression analysis with stepwise selection of established prognostic factors. Factors included in analysis: age, sex, T and N stage, use of concurrent chemotherapy, smoking status and presence of RPLN and HPV status.

**Overall Survival**						
	**Univariate**			Multivariate *		
	**HR**	**95% CI**	***p*-Value**	**HR**	**95% CI**	***p*-Value**
Age	1.03	0.99–1.06	**0.111**			
Sex	0.51	0.20–1.30	**0.158**			
T stage						
T2 vs. T1			0.324			
T3 vs. T1			0.379			
T4 vs. T1	4.59	1.51–13.97	**0.007**	3.67	1.19–11.44	0.024
N stage						
N1 vs. N0			0.942			
N2a vs. N0			0.843			
N2b vs. N0			0.660			
N2c vs. N0			0.427			
N3 vs. N0			0.244			
p16 status (−ve vs. +ve)	2.48	1.19–5.14	**0.014**	2.46	1.11–5.45	0.026
RPLN status (+ve vs. −ve)	2.11	0.97–4.59	**0.060**			
Concurrent chemotherapy (no vs. yes)	2.01	0.95–4.24	**0.066**			
Smoking						
Former vs. never			0.553			
Current vs. never	3.43	1.53–7.67	**0.003**	2.41	1.03–5.65	0.043
**Distant Metastasis–Free Survival**						
	**Univariate**			Multivariate *		
Age			0.37			
Sex			0.64			
T stage						
T2 vs. T1			0.242			
T3 vs. T1	6.24	0.75–51.80	**0.090**			
T4 vs. T1	10.55	1.32–84.43	**0.026**	11.36	1.42–91.04	0.022
N stage						
N1 vs. N0			0.934			
N2a vs. N0			0.95			
N2b vs. N0			0.935			
N2c vs. N0			0.934			
N3 vs. N0			0.93			
p16 status (−ve vs. +ve)	2.84	1.16–6.98	**0.023**	3.09	1.24–7.68	0.015
RPLN status (+ve vs. −ve)	2.41	0.86–6.53	**0.084**			
Concurrent chemotherapy (no vs. yes)	2.07	0.76–5.61	**0.153**			
Smoking						
Former vs. never			0.833			
Current vs. never	3.32	1.15–9.56	**0.026**			

* Factors with *p* < 0.2 in univariate analysis included in multivariate model; *p*-values for these are shown in bold. Hazard ratio and confidence interval shown for factors *p* < 0.2 carried forward into multivariate model. If factors were dropped as nonsignificant from multivariable analysis using backwards likelihood ratios, there were no *p*-values or HR to report. HR = hazard ratio; CI = confidence interval; RPLN = retropharyngeal lymph node.

**Table 4:** Summary of literature reporting outcomes in relation to RPLN status.

		% RPLN+	Imaging	Outcomes (RPLN+ versus RPLN−)	Summary
Current study	n = 402 (p16 available in 226, of which 85% p16+)	10%	PET-CT 100% MRI 84% CT 21%	Five-year LC 81.6% vs. 87.7%, *p* = 0.154 Five-year RC 80.7% vs. 85.4%, *p* = 0.47 Five-year DMFS 73.9% vs. 88.0%, *p* = 0.011 Five-year PFS 62.0% vs. 75.4%, *p* = 0.002 Five-year OS 67.1% vs. 79.1%, *p* = 0.006 Significant on MVA for DMFS, OS Nonsignificant trend for inferior DMFS, OS in p16+ve subgroup	RPLN associated with increased risk DM and inferior OS outcomes
Lin et al., 2019 * [17]	n = 739 N+ and HPV+	9%	RPLN identified by CT in 66%, PET-CT in 7%, CT and PET-CT in 26%, CT and MRI in 1%	Five-year LC 96% vs. 94%, *p* = 0.57 Five-year RC 95% vs. 93%, *p* = 0.47 Five-year DMFS 84% vs. 93%, *p* = 0.033 Five-year OS 74% vs. 87%, *p* = 0.008 Not significant for MVA RP+ associated with inferior DMFS in subgroup of smoking pack years < 10 or concurrent chemotherapy	Difficult to demonstrate association with outcome in HPV+ group. RPLN+ may not be suitable for deintensification
Billfalk-Kelly et al., 2019 [32]	n = 257, T1–2, N1 HPV+ (TNM8 stage 1 only)	8%	CT 67%MRI 33%	DFS: HR 2.62, *p* = 0.021. Not significant for MVA Risk DM: bivariable analysis HR 3.2, *p* = 0.013	RP+ associated with higher risk DM
Baxter et al., 2015 [19]	n = 165 HPV+	10%	100% PET-CT	OR recurrence/death 5.2 No significant association for MVA with outcome	RP+ not independently associated with outcome
Samuels et al., 2015 [12]	n = 185 HPV+	16%	67% PET-CT	Five-year OS 57% vs. 81% (*p* = 0.02) Five-year FFS 63% vs. 80% (*p* = 0.015) Five-year DMFS 70% vs. 91% (*p* = 0.002) No difference local or regional failure MVA: T4, N3 and RP+ independently associated with OS and DMFS	RP+ independent prognostic factor for DM, translating into inferior FFS/OS
Gunn et al., 2013 * [10]	n = 981 (No HPV status)	10%	CT 96% PET 29% MRI 9%	Five-year LC 79% vs. 92%, *p* < 0.01 Five-year RC 80% vs. 93%, *p* < 0.01 Five-year DMFS 66% vs. 89%, *p* < 0.01 Five-year OS 52% vs. 82%, *p* < 0.01 Significant for MVA for LC (*p* = 0.023), DMFS (*p* = 0.003), OS (*p* = 0.001)	RPLN associated with inferior local recurrence and OS, increased risk of DM
Tang et al., 2013 [9]	n = 160 (p16+ve: n = 134)	12%	MRI 46% PET-CT 48% CT 6%	Whole cohort: Two-year OS 71% vs. 89% (HR 2.4, *p* = 0.08) Two-year EFS 71% vs. 81% (HR 2.1, *p* = 0.08) p16+ve only: Two-year OS HR 2.1, *p* = 0.23 Two-year EFS HR 2.0, *p* = 0.16	Nonsignificant trend to worse OS, EFS for RP+ in whole cohort and p16+ subgroup

* Partially overlapping series; RP = retropharyngeal lymph nodes; LC = local control; RC = regional control; DM = distant metastases; DMFS = distant metastases-free survival; EFS = event-free survival; FFS = failure-free survival; OS = overall survival; PFS = progression-free survival; MVA = multivariate analysis; HPV = human papilloma virus; RPLN = retropharyngeal lymph node.
